# Positive childhood experiences in obesity and hypertension among young adults: Associations across adverse childhood experiences levels

**DOI:** 10.1016/j.ajpc.2025.101027

**Published:** 2025-06-07

**Authors:** Vineet Chaudhary, Gagandeep Kaur Walia, Naorem Kiranmala Devi, Kallur Nava Saraswathy

**Affiliations:** a Department of Anthropology, University of Delhi, Delhi 110007, India; b Public Health Foundation of India, Delhi 110030, India

**Keywords:** ACEs, PCEs, BMI, Obesity, Hypertension, Self-esteem

## Abstract

**Background:**

Though the importance of childhood experiences in adult health is increasingly being recognized, the relationship between positive childhood experiences (PCEs) and cardiovascular risk factors remains understudied in low and middle-income countries, including India. This study explored the association of PCE exposure with obesity and hypertension among young adults in Delhi-NCR, India, independently and across different adverse childhood experiences (ACEs) levels.

**Methods:**

A cross-sectional analysis was conducted among 1453 young adults (70.1 % female) recruited from two universities in Delhi-NCR. PCEs were measured using the Benevolent Childhood Experiences scale, and ACEs were assessed with the ACE-International Questionnaire. Obesity and hypertension were measured using standard protocols.

**Results:**

Participants with moderate (6–9) and high (10) PCE exposure showed significantly lower prevalence rates of overweight/obesity (40 % and 41 %, respectively, vs. 57.5 %) and high waist circumference (WC) compared to low (0–5) PCE exposure group (22.9 % and 25.6 %, respectively, vs. 37.7 %). Adjusted logistic regression revealed reduced odds of overweight/obesity and high WC with higher PCE exposure. Specific PCE items, such as self-liking and predictable home routines, were associated with lower obesity risks. No significant association was found between PCE exposure and hypertension. Additionally, a combined analysis of ACE and PCE levels revealed that higher PCEs can mitigate some adverse effects of ACEs on obesity.

**Conclusions:**

Higher PCE exposure may be associated with lower obesity risks and improved anthropometric measures among young adults. Promoting PCEs may help reduce obesity, especially in high-adversity contexts. Interventions enhancing early self-esteem could be crucial in addressing obesity in this population.

**Figure:**
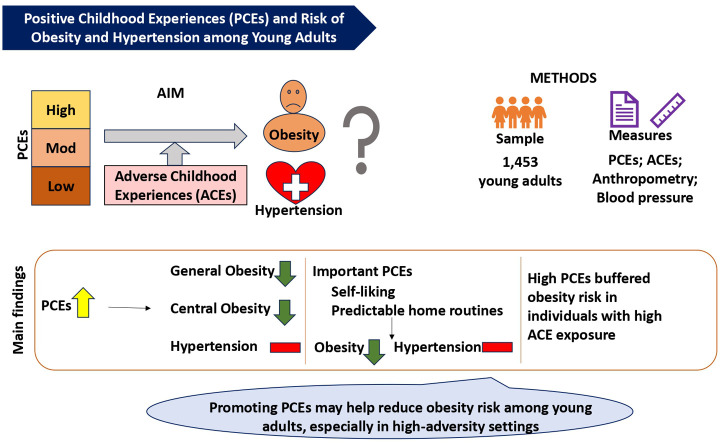
Central Illustration Caption: Higher exposure to Positive Childhood Experiences (PCEs) is linked to lower obesity risk among young adults in Delhi NCR, India, highlighting the protective role of PCEs such as early life self-esteem and stable routines, even in the face of adversity.

## 1 Introduction

The global surge in cardiovascular diseases (CVDs) over recent decades can largely be attributed to the rising prevalence of two major modifiable risk factors, viz., obesity and hypertension [[1], [2], [3]]. In 2022, 43 % of adults were overweight, with 890 million classified as obese, and 1.28 billion adults aged 30–79 years had hypertension [1,3]. India reflects similar trends, with 24 % of women and 22.9 % of men overweight or obese, and hypertension affecting 21.3 % of women and 24 % of men, according to National Family Health Survey (NFHS) - 5 data [4]. Notably, these conditions are increasingly affecting younger populations, including university students, challenging the traditional view of youth as a generally healthy group [5,6]. This demographic is now experiencing a growing burden of non-communicable diseases (NCDs), such as CVDs and mental health issues, as well as communicable conditions like sexually transmitted infections (STIs), highlighting a critical gap in global health strategies and the urgent need to prioritize young adult health [[6], [7], [8]].

Studies during the last two decades have recognized the importance of early life experiences in conditions like obesity and hypertension [[9], [10], [11], [12], [13]]. While the negative health impacts of adverse childhood experiences (ACEs) are well-documented [[9], [10], [11]], there is a growing interest in the positive health outcomes associated with positive childhood experiences (PCEs) [[12], [13], [14], [15], [16], [17], [18]]. PCEs include elements like nurturing parenting, a stable home environment, meaningful beliefs, close relationships with family and friends, a supportive school environment, and support from teachers during the formative years [[14], [15], [16], [17], [18]]. These experiences have consistently been associated with healthy development and enhanced resilience, enabling individuals to cope more effectively with stress and adversity [[18], [19], [20], [21]]. PCEs can be seen as children's assets and resources that serve as key resilience factors [18].

Positive internal assets, such as self-esteem and core beliefs, contribute significantly to long-term health and well-being [18,21,22]. A growing body of research highlights PCEs as robust predictors of favourable health outcomes across the life course [[19], [20], [21]]. Higher levels of PCEs have been consistently associated with reduced risks of mental health disorders, improved sleep quality, safer sexual behaviours, and lower levels of aggression [18]. Further, emerging evidence suggests that exposure to PCEs may offer protection against physical health conditions, including obesity and hypertension [12,13]. Given their potential to mitigate the effects of early adversity and independently promote a range of health outcomes, PCEs are increasingly recognized as vital targets for preventive and therapeutic interventions [12,13,18].

Despite the growing realization regarding the importance of PCEs, fewer attempts have been made to understand the relationship between PCEs and conditions like obesity and hypertension. This is particularly true in the Indian context, where no study so far has attempted to understand the relationship between cumulative PCE exposure and physical health conditions. Although some studies have attempted to understand the effect of one or a few PCE components on physical health, such studies do not allow for a comprehensive comparison between different PCE items and limit the identification of the most important PCE items with respect to different physical health conditions. Considering this gap, the present study attempted to understand the association of cumulative and item-wise PCE exposure with two highly prevalent physical health conditions, namely obesity and hypertension, among young adults in Delhi-National Capital Region (Delhi-NCR), India. The relationship has also been explored across different ACE levels.

## 2 Methods

### 2.1 Study area and participants

The data for this study were derived from a larger ACE study conducted among 1843 college-going young adults (68.1 % females), aged 18–25 years (mean age 19.9 ± 1.89 years), residing in Delhi-NCR. Participants were recruited from various colleges and departments of the University of Delhi, Delhi, and centers and departments of Amity University, Noida, Uttar Pradesh. The inclusion criteria required participants to be Indian citizens, university students, and residents of Delhi-NCR, without self-reported serious physical or mental diseases or disorders. Exclusion criteria included individuals with major physical or mental health conditions, those on long-term medication, pregnant or lactating women, and foreigners. Individuals with prior diagnoses of major physical or mental health conditions were excluded to avoid potential confounding effects of the diagnoses and associated treatments on the relationship between PCEs and the outcome variables.

Of the 1843 individuals in the ACE study, 1453 participants (70.1 % females) who were successfully screened for PCEs, ACEs, and at least one selected outcome variable were included in the present study. The study protocol was approved by the Departmental Ethics Committee (Approval numbers: Ref. No. anth/2022–23/532 and Ref. No. anth/2022–23/14). All participants provided voluntary informed written consent in English prior to their recruitment and data collection.

### 2.2 Data collection and measures

Data collection was performed through in-person interviews with participants. Data on sociodemographic variables like age, gender, educational status, religion, and caste/tribe-based social category were collected using a pretested interview schedule. Participants' socioeconomic status (SES) was determined using the Modified Kuppuswamy Scale [23]. The scale scores range from 0 to 29, with 10 or below indicating lower-class, 11–15 representing lower-middle-class, 16–25 denoting upper-middle-class, and 26–29 indicating upper-class status [23]. For details on caste/tribe-based social categories, refer to [11].

PCE: The Benevolent Childhood Experiences (BCEs) scale was used to assess PCEs [14]. This scale consists of a 10-item checklist of positive experiences during childhood, with each endorsed item contributing one point to the PCE score, resulting in a range from 0 to 10. For categorical analyses, participants were categorized into three groups: 10 PCEs (high PCE), 6–9 PCEs (moderate PCE), and 0–5 PCEs (low PCE) [20].

ACE: Adverse Childhood Experiences International Questionnaire (ACE-IQ)-Frequency Version, which examines exposure to 13 different ACE domains, was used to assess exposure to ACEs [24]. Scoring followed the prescribed guidelines [24]. For categorical analyses, participants were divided into three categories: 0 ACE (no ACE), 1–3 ACEs (moderate ACE), and ≥4 ACEs (high ACE). These cutoffs were based on the meta-analysis by Hughes et al. [25], with 0 ACE as the reference and ≥4 ACEs as the high-risk category. Participants with 1–3 ACEs were grouped into the moderate risk category.

Obesity parameters: Weight, height, waist circumference (WC), and hip circumference (HC) were measured for all participants. Weight was measured using a weighing scale, height with an anthropometer rod, and WC and HC with a steel tape. Body mass index (BMI) was calculated by dividing the weight in kilograms by the square of the height in meters (kg/m²). BMI classifications were: Underweight (<18.5), Normal (18.5–22.9), Overweight (23.0–24.9), and Obese (≥25.0) [26]. A WC of ≥90 cm for males and ≥80 cm for females was considered high [27]. The waist-to-hip ratio (WHR) was determined by dividing WC in cm by HC in cm, with high WHR defined as ≥0.9 for males and ≥0.85 for females [27]. The waist-to-height ratio (WHtR) was calculated by dividing WC in cm by height in cm, with a WHtR of ≥0.5 classified as high [28].

Blood pressure (BP): BP was measured three times for each participant using a digital sphygmomanometer, with a 5-minute interval between each measurement. The average of these three readings was used as the final value. Participants were categorized based on their average systolic BP (SBP) and diastolic BP (DBP) as follows: SBP below 120 mmHg and DBP below 80 mmHg were classified as normal; SBP between 120 and 129 mmHg and DBP below 80 mmHg as elevated; SBP between 130 and 139 mmHg or DBP between 80 and 89 mmHg as Stage 1 hypertension; and SBP ≥140 mmHg or DBP ≥90 mmHg as Stage 2 hypertension [29].

### 2.3 Analytic plan

SPSS version 22 (IBM-SPSS Inc., Chicago, IL) and MS Excel 19 were used for statistical analyses. Descriptive statistics (frequencies, percentages, means, and standard deviations) of dependent variables (obesity parameters and blood pressure/hypertension) were calculated for low, moderate, and high PCE exposure categories. Differences in prevalence and mean values were assessed using chi-square tests and ANOVA, respectively. Linear regression analysed the association between PCE scores and health outcomes. Logistic regression calculated the odds of obesity and hypertension at higher PCE levels compared to low PCE level. Linear and logistic regression models were adjusted for age, sex, education, and socioeconomic status, with additional adjustments for social category in obesity models (as social category was found to influence obesity, data not shown).

Further, the prevalence of obesity and hypertension, expressed as frequencies and percentages, was computed across exposed and unexposed groups for each PCE item. Differences in prevalence were assessed using chi-square tests. Adjusted logistic regression analyses calculated the odds of studied health outcomes for individuals exposed to individual PCE items compared to those unexposed.

To understand the relationship between ACE/PCE and studied health outcomes in the context of each other, the sample was stratified by ACE exposure to explore the relationship between PCE and outcome variables at specific ACE levels, and vice versa. This approach helped understand how PCEs might influence health outcomes in high- versus low-adversity contexts, highlighting different needs based on ACE exposure levels. Variations in levels of obesity parameters and blood pressure/hypertension were reported as prevalence and mean scores across ACE subcategories stratified by PCE exposure and PCE subcategories stratified by ACE exposure. Differences in prevalence and mean values were assessed using chi-square tests and ANOVA, respectively. Adjusted linear regression determined the association between independent variables (PCE and ACE) and dependent variables (studied outcomes) within each stratified group. Pearson correlation analysis was employed to assess the strength of linear relationships between continuous variables. Missing data was handled using pairwise deletion. A *p*-value of less than 0.05 was considered statistically significant.

## 3 Results

### 3.1 General characteristics of the study sample

Of the total participants, 70.1 % were females, and 51.9 % were below 20 years of age (Supplementary Table 1). Most participants were undergraduates (87.9 %) and middle class (upper middle, 37.4 %; lower middle, 20.3 %). The predominant religion was Hinduism (84.5 %), followed by Islam (10.4 %) and other religions (5.1 %). Social categories included Unreserved (67 %), Other Backward Classes (OBC; 20 %), Scheduled Castes (SC; 10.6 %), and Scheduled Tribes (ST; 2.4 %) (Supplementary Table 1).

### 3.2 Physical health outcomes in various PCE categories: descriptive statistics

The prevalence of studied physical health outcomes was examined across various PCE categories. The analysis showed a significant decrease in overweight/obesity from 57.5 % in the 0–5 (low) PCE category to 40 % and 41 % in the 6–9 (moderate) and 10 (high) PCE categories, respectively (Table 1). High WC prevalence was also significantly higher in the low PCE category (37.7 %) compared to the moderate and high PCE categories (22.9 % and 25.6 %, respectively). Again, the prevalence of high WHR and high WHtR was higher in the low PCE category than in the higher (6–9 and 10) PCE categories; however, the trend was not statistically significant. Surprisingly, the proportion of individuals with normal BP was significantly higher in the low PCE category (70.5 %) than in the moderate and high PCE categories (60.9 % and 60.3 %, respectively) (Table 1).

**Table 1:** Prevalence of obesity and hypertension in relation to PCEs.

Physical health outcomes	Status	PCEs			*p*-value
		0–5 (Low)	6–9 (Moderate)	10 (High)	
		N ( %)	N ( %)	N ( %)	
BMI	Normal	36 (34)	276 (38.2)	254 (41)	0.002*
	Underweight	9 (8.5)	157 (21.7)	112 (18.1)	
	Overweight/obese	61 (57.5)	289 (40)	254 (41)	
WC	Normal	66 (62.3)	556 (77.1)	458 (74.4)	0.004*
	High	40 (37.7)	165 (22.9)	158 (25.6)	
WHR	Normal	76 (71.7)	554 (76.8)	490 (79.7)	0.143
	High	30 (28.3)	167 (23.2)	125 (20.3)	
WHtR	Normal	64 (60.4)	486 (67.6)	408 (66.2)	0.335
	High	42 (39.6)	233 (32.4)	208 (33.8)	
BP	Normal	74 (70.5)	434 (60.9)	369 (60.3)	0.036*
	Elevated	6 (5.7)	47 (6.6)	52 (8.5)	
	HTN Stage 1	20 (19)	190 (26.6)	135 (22.1)	
	HTN Stage 2	5 (4.8)	42 (5.9)	56 (9.2)	

⁎ Significant at *p*-value < 0.05; *N* = Count; % = Column Wise Percentage; PCEs = Positive Childhood Experiences; BMI = Body Mass Index; WC = Waist Circumference; WHR = Waist-to-Hip Ratio; WHtR = Waist-to-Height Ratio; BP = Blood Pressure; HTN = Hypertension

**Fig. 1:**
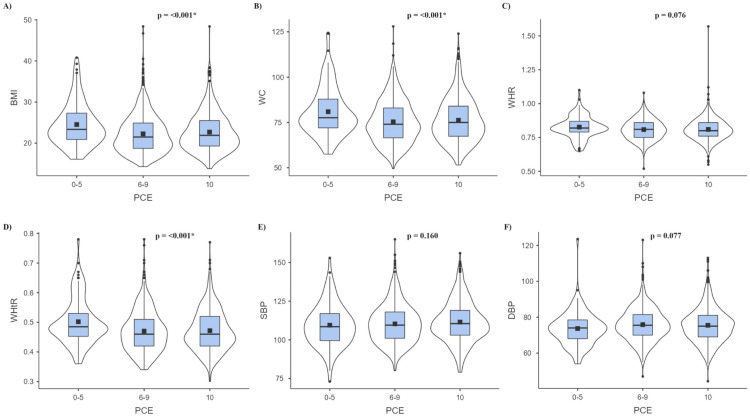
Mean levels of A) BMI, B) WC, C) WHR, D) WHtR, E) SBP, F) DBP in PCE categories. *Significant at *p*-value<0.05; PCE = Positive Childhood Experience; BMI = Body Mass Index; WC = Waist Circumference; WHR = Waist-to-Hip Ratio; WHtR = Waist-to-Height Ratio; SBP = Systolic Blood Pressure; DBP = Diastolic Blood Pressure.

Coming to mean values, mean BMI, WC, and WHtR were significantly higher in the low PCE category compared to the higher PCE categories (Fig. 1). In terms of BP variables, while the mean SBP and mean DBP were lower in the low PCEs category than in the higher PCEs categories, these differences were not found to be statistically significant (Fig. 1).

### 3.3 Variations in anthropometric and BP measures with PCE score

Adjusted linear regression analysis explored the relationship between PCE scores and studied anthropometric and BP measures. The findings revealed that higher levels of PCEs were significantly associated with lower BMI, smaller WC, and lower WHtR (Supplementary Table 2). Further, while the trend suggested a lower WHR with an increased PCE score, this relationship did not attain statistical significance. The PCE score did not significantly predict SBP or DBP (Supplementary Table 2).

### 3.4 Cumulative PCE exposure and odds of obesity and hypertension

With the low PCE category as the reference, the odds of overweight/obesity and hypertension, in higher PCE categories were computed. The analyses revealed that exposures to moderate and high PCE were associated with significantly reduced odds of overweight/obesity (Table 2). Again, compared to the low PCE category, the odds of high WC were found to be lower in the moderate and high PCE categories (Table 2). However, higher PCE exposures were not found to be significantly associated with hypertension (Table 2).

**Table 2:** Association between cumulative PCE exposure and obesity and hypertension.

PCE categories	BMI		WC	WHR	WHtR	BP
	Underweight	Overweight/ obese	High	Medium	High	High (stage 1 + 2)
	OR^a^ (95 % CI)	OR^a^ (95 % CI)	OR^a^ (95 % CI)	OR^a^ (95 % CI)	OR^a^ (95 % CI)	OR^b^ (95 % CI)
0–5	Reference					
6–9	1.52 (0.65–3.54)	0.55 (0.32–0.94)	0.62 (0.37–1.02)	0.88 (0.52–1.49)	0.87 (0.53–1.41)	1.27 (0.76–2.11)
*p*-value	0.332	0.030*	0.062	0.635	0.562	0.362
10	1.14 (0.49–2.68)	0.45 (0.26–0.78)	0.61 (0.37–1.02)	0.64 (0.37–1.09)	0.76 (0.47–1.24)	1.21 (0.72–2.02)
*p*-value	0.758	0.004*	0.058	0.100	0.276	0.470

⁎ Significant at *p*-value < 0.05; PCEs = Positive Childhood Experiences; OR = Odds Ratio; CI = Confidence Interval; BMI = Body Mass Index; WC = Waist Circumference; WHR = Waist-to-Hip Ratio; WHtR = Waist-to-Height Ratio; BP = Blood Pressure; stage 1 + 2 = Hypertension Stage 1 + 2.

a adjusted for age, sex, education, socioeconomic status, and social category.

b adjusted for age, sex, education, socioeconomic status.

### 3.5 Distribution of obesity, and hypertension in groups exposed and not exposed to PCE items

To understand the impact of exposure to specific PCE items on physical health, the distribution of obesity and hypertension was examined in groups exposed and unexposed to individual PCE items. The analysis revealed that, of the 10 PCE items, the proportions of individuals with overweight/obesity, high WC, high WHR, and high WHtR were significantly lower in the exposed group than in the unexposed group for 2, 3, 1, and 1 PCE item(s), respectively (Supplementary Table 3). Notably, the proportion of individuals with any type of obesity was significantly lower in the group that ‘liked themselves/felt comfortable with themselves’ than in the group that did not. No significant difference in the distribution of hypertension was found between the exposed and unexposed groups for any of the PCE items (Supplementary Table 4).

### 3.6 Exposure to different PCE items and odds of overweight/obesity, and hypertension

Adjusted logistic regression analysis was performed to compute the odds of overweight/ obesity, and hypertension among those exposed to different PCE items with their unexposed counterparts as a reference. Of the 10 PCE items, when compared to the unexposed group, the exposed group was found to be at significantly lower risk of overweight/obesity, high WC, and high WHR for 2, 1, and 2 items, respectively (Table 3). Further, PCE-exposed individuals were found to be at lower risk of overweight/obesity for three more items, with borderline significance. It is worth noting that feeling comfortable with oneself was found to be associated with reduced odds of both general and central obesity (high WHR). However, individual PCE items did not show a significant association with WHtR, or hypertension (Table 3).

**Table 3:** Exposure to PCE items and odds for obesity, and hypertension (with absence of respective PCEs items as the reference).

PCE items		BMI		WC	WHR	WHtR	BP
		Underweight	Overweight/obese	High	High	High	High (stage 1 + 2)
		OR^a^ (95 % CI)	OR^a^ (95 % CI)	OR^a^ (95 % CI)	OR^a^ (95 % CI)	OR^a^ (95 % CI)	OR^b^ (95 % CI)
Did you have at least one caregiver with whom you felt safe?		0.75 (0.44–1.28)	0.65 (0.42–1.01)	0.84 (0.54–1.29)	1.11 (0.7–1.76)	0.76 (0.51–1.12)	0.95 (0.64–1.4)
	p	0.289	0.056	0.428	0.662	0.166	0.781
Did you have at least one good friend?		1.05 (0.61–1.81)	0.66 (0.43–1.01)	0.77 (0.5–1.18)	0.88 (0.57–1.38)	0.84 (0.57–1.26)	0.98 (0.66–1.44)
	p	0.867	0.058	0.229	0.586	0.405	0.909
Did you have ‘beliefs’ that gave you comfort?		1.18 (0.68–2.05)	0.73 (0.48–1.11)	0.62 (0.42–0.93)	1.18 (0.75–1.86)	0.84 (0.58–1.24)	0.87 (0.6–1.27)
	p	0.549	0.146	0.021*	0.466	0.387	0.470
Did you have enjoyment at school?		1.39 (0.75–2.59)	0.71 (0.47–1.07)	0.74 (0.49–1.1)	1 (0.65–1.55)	0.83 (0.56–1.21)	1.28 (0.86–1.9)
	p	0.295	0.105	0.137	0.998	0.325	0.218
Did you have at least one teacher that cared?		1.08 (0.65–1.8)	0.9 (0.6–1.34)	0.94 (0.62–1.41)	0.73 (0.49–1.1)	0.95 (0.66–1.39)	0.94 (0.66–1.36)
	p	0.765	0.605	0.759	0.134	0.802	0.760
Did you have good neighbours?		1.11 (0.75–1.65)	0.75 (0.54–1.02)	0.9 (0.65–1.24)	0.72 (0.52–0.99)	0.85 (0.63–1.13)	1.03 (0.77–1.37)
	p	0.596	0.069	0.524	0.045*	0.265	0.865
Did you have an adult in your life (other than the person from question 1) who could provide you with support or advice?		0.74 (0.49–1.11)	1.02 (0.71–1.46)	0.9 (0.63–1.28)	0.85 (0.59–1.23)	1.03 (0.74–1.43)	1.18 (0.85–1.64)
	p	0.145	0.927	0.554	0.397	0.879	0.333
Did you have opportunities to have a good time?		0.88 (0.48–1.63)	0.88 (0.53–1.47)	1.21 (0.71–2.07)	0.89 (0.54–1.49)	1.32 (0.81–2.15)	1.36 (0.84–2.2)
	p	0.691	0.635	0.486	0.671	0.258	0.212
Did you like yourself or felt comfortable with yourself?		0.91 (0.49–1.68)	0.56 (0.36–0.86)	0.77 (0.52–1.14)	0.65 (0.43–0.97)	0.77 (0.53–1.12)	1.41 (0.94–2.11)
	p	0.759	0.009*	0.194	0.037*	0.175	0.092
Did you have predictable home routine, like regular meals and a regular bedtime?		0.81 (0.54–1.19)	0.7 (0.5–0.97)	0.79 (0.56–1.09)	0.85 (0.6–1.19)	0.86 (0.63–1.16)	0.98 (0.73–1.32)
	p	0.280	0.032*	0.154	0.332	0.316	0.914

⁎ Significant at *p*-value < 0.05; *p* = *p*-value; PCEs = Positive Childhood Experiences; OR = Odds Ratio; CI = Confidence Interval; BMI = Body Mass Index; WC = Waist Circumference; WHR = Waist-to-Hip Ratio; WHtR = Waist-to-Height Ratio; BP = Blood Pressure; stage 1 + 2= Hypertension Stage 1 + 2.

a adjusted for age, sex, education, socioeconomic status, and social category.

b adjusted for age, sex, education, socioeconomic status.

### 3.7 Mean anthropometric and BP measures across ACE subcategories stratified for PCE level and PCE subcategories stratified for ACE levels

Variations in the mean anthropometric and BP measures across ACE subcategories stratified for PCE level and PCE subcategories stratified for ACE levels were examined. For a given PCE level, there were generally no statistically significant differences in mean values across ACE subcategories for any of the studied anthropometric and BP measures (Table 4). Exceptions were a statistically significant increase in mean BMI and a decrease in mean DBP through 0 (no) to ≥4 (high) ACE subcategories in the 6–9 (moderate) PCE category and the 10 (high) PCE category, respectively.

**Table 4:** Mean anthropometric and blood pressure measures in ACE subcategories stratified for PCE levels and PCE subcategories stratified for ACE levels.

Stratification	Subcategory	BMI	WC	WHR	WHtR	SBP	DBP
		Mean ± SD	Mean ± SD	Mean ± SD	Mean ± SD	Mean ± SD	Mean ± SD
0–5 PCEs	0 ACE	24.76 ± 8.07	85.5 ± 14.46	0.84 ± 0.08	0.51 ± 0.1	111.33 ± 18.01	75.39 ± 10.54
	1–3 ACEs	23.89 ± 5.23	79.19 ± 13.56	0.82 ± 0.08	0.49 ± 0.08	109.3 ± 14.39	72.92 ± 11.28
	≥ 4 ACEs	25.22 ± 4.79	82.26 ± 13.24	0.83 ± 0.08	0.51 ± 0.07	109.41 ± 12.75	74.28 ± 6.95
	*p*-value	0.457	0.348	0.524	0.458	0.935	0.693
6–9 PCEs	0 ACEs	21.53 ± 4.35	73.91 ± 11.93	0.81 ± 0.08	0.46 ± 0.06	110.57 ± 13.15	76.42 ± 8.2
	1–3 ACEs	22.34 ± 4.69	75.48 ± 11.52	0.81 ± 0.08	0.47 ± 0.07	110.65 ± 12.51	76.04 ± 8.92
	≥ 4 ACEs	22.93 ± 5.11	76.67 ± 12.37	0.81 ± 0.07	0.47 ± 0.08	109.08 ± 12.55	74.67 ± 9.77
	*p*-value	0.031*	0.119	0.931	0.343	0.436	0.200
10 PCEs	0 ACE	22.49 ± 4.98	75.29 ± 12.33	0.8 ± 0.07	0.47 ± 0.07	111.67 ± 12.99	76.85 ± 9.37
	1–3 ACEs	22.85 ± 4.63	76.91 ± 12.38	0.82 ± 0.09	0.48 ± 0.07	111.66 ± 13.89	75.15 ± 9.84
	≥ 4 ACEs	22.65 ± 4.12	75.77 ± 11.16	0.81 ± 0.07	0.46 ± 0.06	110.07 ± 12.6	72.78 ± 8.32
	*p*-value	0.682	0.300	0.181	0.172	0.659	0.006*
0 ACE	0–5 PCEs	24.76 ± 8.07	85.5 ± 14.46	0.84 ± 0.08	0.51 ± 0.1	111.33 ± 18.01	75.39 ± 10.54
	6–9 PCEs	21.53 ± 4.35	73.91 ± 11.93	0.81 ± 0.08	0.46 ± 0.06	110.57 ± 13.15	76.42 ± 8.2
	10 PCEs	22.49 ± 4.98	75.29 ± 12.33	0.80 ± 0.07	0.47 ± 0.07	111.67 ± 12.99	76.85 ± 9.37
	*p*-value	0.052	0.038*	0.315	0.166	0.726	0.839
1–3 ACEs	0–5 PCEs	23.89 ± 5.23	79.19 ± 13.56	0.82 ± 0.08	0.49 ± 0.08	109.3 ± 14.39	72.92 ± 11.28
	6–9 PCEs	22.34 ± 4.69	75.48 ± 11.52	0.81 ± 0.08	0.47 ± 0.07	110.65 ± 12.51	76.04 ± 8.92
	10 PCEs	22.85 ± 4.63	76.91 ± 12.38	0.82 ± 0.09	0.48 ± 0.07	111.66 ± 13.89	75.15 ± 9.84
	*p*-value	0.049*	0.054	0.542	0.095	0.375	0.060
≥4 ACEs	0–5 PCEs	25.22 ± 4.79	82.26 ± 13.24	0.83 ± 0.08	0.51 ± 0.07	109.41 ± 12.75	74.28 ± 6.95
	6–9 PCEs	22.93 ± 5.11	76.67 ± 12.37	0.81 ± 0.07	0.47 ± 0.08	109.08 ± 12.55	74.67 ± 9.77
	10 PCEs	22.65 ± 4.12	75.77 ± 11.16	0.81 ± 0.07	0.46 ± 0.06	110.07 ± 12.6	72.78 ± 8.32
	*p*-value	0.010*	0.012*	0.074	0.002*	0.871	0.362

⁎ Significant at *p*-value < 0.05; ACE = Adverse Childhood Experience; PCE = Positive Childhood Experience; SD = Standard Deviation; BMI = Body Mass Index; WC = Waist Circumference; WHR = Waist-to-Hip Ratio; WHtR = Waist-to-Height Ratio; SBP = Systolic Blood Pressure; DBP = Diastolic Blood Pressure.

Coming to variation in mean anthropometric and BP measures through PCE subcategories for a given level of ACEs, the findings indicated a general decrease in mean BMI and mean WC from 0–5 low to 10 (high) PCE subcategories for a given ACEs level (Table 4). This trend was particularly pronounced in the ≥4 (high) ACEs category. Further, mean WHR and mean WHtR also exhibited a decrease from 0–5 (low) to 10 (high) PCE subcategories in the ≥4 (high) ACEs category. All these observed trends either reached statistical significance or approached significance. No significant variations in mean SBP and mean DBP across PCE subcategories were observed for any of the ACE levels. For the prevalence of obesity and hypertension in ACE subcategories stratified for PCE levels and PCE subcategories stratified for ACE levels refer to Supplementary Table 5.

### 3.8 Association of PCEs and ACEs with studied health outcomes in the context of one another

Adjusted linear regression analyses were undertaken to understand the variations in anthropometric and BP measures with ACE scores for given PCE levels and with PCE scores for given ACE levels. In general, ACE score was not found to be significantly associated with anthropometric and blood pressure measures for any of the PCE levels, except for a significant positive association with BMI in the 6–9 (moderate) PCE category and a significant inverse association with DBP in the 10 (high) PCE category (Table 5).

**Table 5:** Stratified linear regression between ACE/ PCE scores and health parameters in context of each other.

Stratification	Dependent variable	R	R^2^	β (Unstandardized)	Std. Error	Beta (Standardized)	t	*p*-value
Independent variable: ACE score								
0–5 PCEs	BMI^a^	0.357	0.128	0.256	0.237	0.120	1.078	0.285
	WC^a^	0.365	0.133	0.333	0.615	0.060	0.542	0.590
	WHR^a^	0.346	0.12	0.002	0.004	0.073	0.649	0.518
	WHtR^a^	0.301	0.091	0.003	0.004	0.108	0.952	0.344
	SBP^b^	0.582	0.339	0.049	0.531	0.009	0.092	0.927
	DBP^b^	0.223	0.05	0.146	0.456	0.038	0.321	0.749
6–9 PCEs	BMI^a^	0.399	0.159	0.234	0.091	0.099	2.577	0.010*
	WC^a^	0.513	0.263	0.330	0.213	0.056	1.550	0.122
	WHR^a^	0.508	0.258	−0.001	0.001	−0.034	−0.935	0.350
	WHtR^a^	0.377	0.142	0.002	0.001	0.045	1.167	0.244
	SBP^b^	0.481	0.232	−0.190	0.234	−0.030	−0.812	0.417
	DBP^b^	0.144	0.021	−0.079	0.185	−0.018	−0.427	0.670
10 PCEs	BMI^a^	0.289	0.084	0.017	0.132	0.005	0.127	0.899
	WC^a^	0.407	0.166	−0.133	0.323	−0.017	−0.411	0.681
	WHR^a^	0.390	0.152	0.000	0.002	−0.007	−0.178	0.859
	WHtR^a^	0.297	0.088	−0.001	0.002	−0.017	−0.393	0.695
	SBP^b^	0.456	0.208	−0.304	0.347	−0.036	−0.878	0.380
	DBP^b^	0.194	0.038	−0.705	0.279	−0.113	−2.524	0.012*
Independent variable: PCE scores								
0 ACE	BMI^a^	0.298	0.089	−0.143	0.195	−0.040	−0.733	0.464
	WC^a^	0.422	0.178	−0.525	0.464	−0.058	−1.131	0.259
	WHR^a^	0.445	0.198	−0.003	0.003	−0.051	−0.996	0.320
	WHtR^a^	0.275	0.075	−0.003	0.003	−0.058	−1.053	0.293
	SBP^b^	0.458	0.21	0.256	0.465	0.027	0.551	0.582
	DBP^b^	0.169	0.029	0.378	0.359	0.057	1.054	0.293
1–3 ACEs	BMI^a^	0.382	0.146	0.013	0.104	0.004	0.121	0.903
	WC^a^	0.479	0.23	−0.128	0.249	−0.018	−0.514	0.607
	WHR^a^	0.425	0.181	−0.001	0.002	−0.015	−0.423	0.672
	WHtR^a^	0.357	0.127	−0.001	0.002	−0.012	−0.323	0.747
	SBP^b^	0.459	0.211	0.052	0.269	0.007	0.195	0.846
	DBP^b^	0.109	0.012	0.058	0.217	0.010	0.266	0.790
≥4 ACEs	BMI^a^	0.429	0.184	−0.653	0.143	−0.304	−4.572	<0.001*
	WC^a^	0.509	0.259	−1.397	0.349	−0.255	−4.000	<0.001*
	WHR^a^	0.519	0.269	−0.005	0.002	−0.159	−2.497	0.013*
	WHtR^a^	0.447	0.2	−0.009	0.002	−0.280	−4.226	<0.001*
	SBP^b^	0.490	0.24	−0.407	0.331	−0.074	−1.231	0.219
	DBP^b^	0.114	0.013	−0.143	0.268	−0.036	−0.532	0.595

⁎ Significant at *p*-value < 0.05; PCEs = Positive Childhood Experiences; ACEs = Adverse Childhood Experiences; BMI = Body Mass Index; WC = Waist Circumference; WHR = Waist-to-Hip Ratio; WHtR = Waist-to-Height Ratio; SBP = Systolic Blood Pressure; DBP = Diastolic Blood Pressure.

a adjusted for age, sex, education, socioeconomic status, and social category.

b adjusted for age, sex, education, socioeconomic status; *R*= Correlation Coefficient; R^2^= Coefficient of Determination; β= regression coefficient; *t* = *t*-statistic.

Coming to variation in anthropometric and BP measures with variation in PCE scores for given ACE levels, the linear regression analysis revealed no significant association between PCE scores and studied anthropometric and BP measures in the 0 (no) ACEs and 1–3 (moderate) ACEs categories. Nevertheless, the PCE score exhibited a significant inverse association with BMI, WC, WHR, and WHtR in the ≥4 (high) ACEs category (Table 5). For correlation patterns of ACEs score stratified for PCE level and PCE score stratified for ACEs level with anthropometric and BP measures, refer to Supplementary Table 6.

## 4 Discussion

Higher PCE exposure was associated with a lower prevalence of overweight/obesity and high WC, along with reduced mean BMI, WC, and WHtR. Regression analyses confirmed an inverse relationship between PCE scores and adiposity measures, as well as a decreased risk of overweight/obesity and high WC (borderline significance). These findings suggest that moderate to high PCE exposure may help protect against weight gain and the development of both general and central obesity. Only a few studies thus far have attempted to understand the relationship between cumulative PCE exposure and adult obesity [12,17,30]. Consistent with the observations of the present study, previous studies have reported high PCE exposure to be protective against the risk of adult overweight/obesity [12], and childhood overweight/obesity [30]; however, no significant association has also been reported [17]. Apart from obesity, high PCE exposure has been found to be protective against other physical health conditions/, for instance, asthma [31], sleep problems [32], digestive system diseases, chronic headaches and neck/back pain [12], and cognitive functioning [33].

Coming to mechanisms, PCEs have been shown to have both promotive, where PCEs independently promote positive health outcomes regardless of the level of ACE exposure, and protective effect, where PCEs buffer against the detrimental effects of ACEs on health [18,34]. For instance, PCEs can promote the adoption of a healthier lifestyle and thereby reduce the risk of obesity [12,18]. High PCE exposure has also been found to be associated with better health-related behaviours during adulthood in terms of low risk of substance use [16], and increased physical activity [12]; however, no associations have also been reported [17,35]. Alternatively, PCEs can reduce the tendency to engage in risky health behaviours as well as the risk of poor mental health among ACE-exposed individuals [18], both of which are independent risk factors of obesity [36,37].

Besides behavioural and mental health-related factors, some lines of evidence hint toward the involvement of biological pathways in the promotive/protective effect of PCEs. In a recent study, increased positive parenting was found to be associated with slower epigenetic/biological aging among children who experienced high levels of adversity [38]. Further, studies have indicated that social factors like positive parenting and peer support can potentially buffer children and adolescents from dysregulation of the Hypothalamic-Pituitary-Adrenal (HPA) axis [39,40]. Based on emerging evidence, it can be suggested that slower epigenetic aging could potentially result in a more regulated metabolic profile, thereby reducing the risk of excessive weight gain. Additionally, buffering the dysregulation of the HPA axis might mitigate the effects of chronic stress, which is often associated with unhealthy behaviours and weight gain. How PCEs may promote better health outcomes through biological pathways, viz, through HPA-axis or epigenetic pathways, is a matter of curiosity [38,39], and should be investigated in future studies.

Regarding individual PCE items, of the 10 PCE items, two (liking oneself/feeling comfortable with oneself and predictable home routine) were associated with a significantly reduced risk of general overweight/obesity, and three (having beliefs that gave comfort, having good neighbours, and liking oneself/feeling comfortable with oneself) were associated with a significantly reduced risk of central obesity. It is worth underscoring that liking oneself /feeling comfortable with oneself during childhood was protective against both general and central obesity in adult life. The findings of the present study with respect to liking oneself or having positive self-esteem during childhood and adult obesity are consistent with some of the previous studies [41,42]. Supplementary to these reports, a recent paper exploring the longitudinal relationship between self-esteem and obesity in a general adult population sample also found a significant inverse relationship [43].

It has been reported that individuals with low self-esteem discount the future more or participate less in future-oriented planning, leading to increased food intake and reduced physical activity levels, eventually resulting in weight gain and obesity [43]. Conversely, it can be suggested that those children with high self-esteem may be more future-oriented and inclined towards a healthier lifestyle. It has also been observed that self-esteem during early childhood may be more consequential in terms of adult health outcomes than that during mid or late adolescence [41,43]. Therefore, intervention to improve early self-esteem could be particularly beneficial for long-term health outcomes.

In the present study, PCE exposure was not found to be significantly associated with hypertension. Further, none of the 10 PCE items were found to be associated with hypertension. Similar to the observations of this study, two recent studies on the relationship between PCEs and different physical health outcomes reported no significant relationship between PCE exposure and adult cardiovascular diseases (including hypertension as one component) [12,13]. In another study examining the relationship between PCEs and midlife cardiovascular health, differences in the prevalence of hypertension across PCE quartiles were not found to be statistically significant [44]. Further, a recent study by Huang et al. also did not find PCE exposure protective against adult hypertension [31].

Overall, PCE exposure appears to have a limited role in reducing the risk of adult hypertension. These observations are rather surprising, especially since PCE exposure has been reported to be associated with better adult physical health outcomes [13], and ideal cardiovascular health metrics in midlife [44]. It is also counterintuitive given the inverse association between PCE exposure and the risk of adult overweight/obesity in the present study. Nevertheless, these observations are consistent with the limited existing literature [12,13,44]. One possible explanation for the lack of a significant association between PCEs and blood pressure parameters, despite their association with obesity and related anthropometric measures, may lie in the timing of these health outcomes. While PCEs promote resilience, stress regulation, and healthier lifestyle choices that can help prevent excessive weight gain even in early life, their effects on cardiovascular markers such as hypertension may take longer to emerge. This is particularly relevant given that the study sample comprises young adults. Existing literature suggests that the physiological consequences of ACEs, including elevated blood pressure, often become more apparent after the age of 30 years [11]. Therefore, it is plausible that the protective effects of PCEs on blood pressure may also manifest more clearly later in life. Given the limited literature on the relationship between PCEs and hypertension, it remains difficult to generalize the findings. Further research, especially longitudinal follow-up studies, is warranted to better understand this delayed protective trajectory.

Next, to examine the association between ACEs and adult physical health outcomes across PCE levels, the sample was stratified into 0–5 (low), 6–9 (moderate), and 10 (high) PCE exposure levels. Interestingly, ACE exposure was not found to be associated with any of the studied physical health parameters across any of the PCE levels, except for BMI in the moderate PCE category and DBP in the high PCE category. Put differently, for a particular level of PCE exposure, ACEs failed to account for the variances observed in obesity or BP parameters, except for BMI and DBP in the moderate and high PCE categories, respectively. This suggests that PCE exposure may play a predominant role in shaping the relationship between ACE and obesity parameters, particularly in low or high PCE contexts. In low PCE contexts, obesity may be promoted due to the absence of PCEs, independent of ACE exposure. In contrast, in high PCE contexts, PCE may counterbalance the risk conferred by ACE exposure. The negative association between ACE exposure and DBP in a high PCE context may be indicative of basal cardiovascular hypoactivation [45].

Further, to understand the association between PCE exposure and adult physical health outcomes across ACE levels, the sample was stratified into 0 (no), 1–3 (moderate), and ≥4 (high) ACE levels. In this analysis, PCE exposure was found to be inversely associated with BMI, WC, WHR, and WHtR only in the ≥4 (high) ACE category but not in the moderate or no ACE category. Similar observations were reported in a recent study where exposure to PCEs was associated with a reduced likelihood of being overweight/obese among children who had experienced high ACEs but not among those who had experienced zero to one ACEs [30]. In another study, higher family resilience reduced the odds of being in a higher-weight category among ACE-exposed children but not among ACE-unexposed children [46]. However, conflicting findings, where PCEs were more strongly associated with some favourable outcomes in low adversity context than in the high adversity context, have also been reported [17,35].

The findings of the present study point towards the protective role of PCE with respect to obesity in the context of high adversity. However, PCE exposure does not appear to be independently promoting healthy levels of body weight, as indicated by the lack of association between PCE exposure and obesity parameters in low adversity contexts. Overall, the stratified analyses hint towards the protective but not promotive role of PCEs on the studied physical health parameters in the present sample. Together, these findings suggest that interventions to promote PCEs can positively affect adult obesity parameters, especially in high-adversity contexts. Improving early self-esteem can be particularly important in this regard.

Some of the limitations of the present study should be acknowledged. An important limitation is the higher proportion of females compared to males in the sample. To compensate for this, all regression models have been adjusted for sex. Additionally, the sample comprises university students, which may restrict the generalizability of the results to other demographic groups. Furthermore, while the ACE-IQ and BCE scales are widely used to assess adverse and positive childhood experiences, respectively, they may not fully capture these experiences within the Indian context, highlighting the need for India-specific assessment tools. Moreover, despite the apparent precedence of ACEs/PCEs over adult health outcomes, a causal relationship cannot be confidently inferred without information on the timing of the exposures and outcomes. Therefore, longitudinal studies are necessary to deepen our understanding of this relationship.

## 5 Conclusions

The present study highlights the protective effects of PCE on obesity parameters among young adults. Moderate or high exposure to PCEs was found to significantly reduce the prevalence of overweight/obesity and high WC, and to lower the mean BMI. Individuals with higher PCE exposure had a lower risk of general and central obesity compared to those with low PCE exposure. Specific PCE items such as liking oneself, having a predictable home routine, comforting beliefs, and supportive neighbours were linked to reduced risks of obesity. In contrast, no significant association was found between cumulative PCE exposure and hypertension. Regarding the combined effect of ACEs and PCE exposures, PCE exposure might mitigate the negative impact of ACEs on obesity. These findings suggest that promoting PCEs can have a beneficial impact on reducing obesity, particularly in high-adversity contexts. Interventions aimed at improving early self-esteem and providing supportive environments could be particularly important in addressing obesity among youth.

## Funding

This work was supported by the Institute of Eminence, University of Delhi, under the grant Ref. No./IoE/2021/12/FRP and the Indian Council of Medical Research under the grant 5/4/8-22/CD/KNS/2021-NCD-II.

## Conflict of interest

The author declared no potential conflicts of interest.

## Ethics approval

The study procedures were carried out in accordance with the Declaration of Helsinki. The study protocol was approved by the Departmental Ethics Committee, Department of Anthropology, University of Delhi (Approval number: Ref. No. Anth/2022–23/532, Ref. No. Anth/2022–23/14, and Ref. No. Anth/2022–23/524).

## Informed consent

Informed written consent, typed in English, was obtained from each participant prior to their recruitment.

## Consent for publication

Not applicable.

## CRediT authorship contribution statement

**Vineet Chaudhary:** Writing – original draft, Methodology, Investigation, Formal analysis, Data curation, Conceptualization. **Gagandeep Kaur Walia:** Writing – review & editing, Methodology, Formal analysis. **Naorem Kiranmala Devi:** Writing – review & editing, Methodology. **Kallur Nava Saraswathy:** Writing – review & editing, Validation, Supervision, Resources, Methodology, Conceptualization.

## Declaration of competing interest

The authors declare that they have no known competing financial interests or personal relationships that could have appeared to influence the work reported in this paper.

## Appendix Supplementary materials

Image, application 1
