# Supplementary material for: Positive childhood experiences in obesity and hypertension among young adults: Associations across adverse childhood experiences levels
Source: Am J Prev Cardiol. 2025 Jun 7;23:101027. doi: 10.1016/j.ajpc.2025.101027 (PMC12221651; doi:10.1016/j.ajpc.2025.101027)
Supplement: Supplementary file 1 [file mmc1.docx]

Supplementary Table 1: General characteristics of the study sample

| Variables | | Status | N (%) |
| --- | --- | --- | --- |
| Sex | Male | | 434 (29.9) |
|  | Female | | 1019 (70.1) |
| Age group (in years) | 18-19 | | 754 (51.9) |
|  | 20-21 | | 452 (31.1) |
|  | 22-23 | | 189 (13.0) |
|  | 24-25 | | 58 (4.0) |
| Education | UG | | 1277 (87.9) |
|  | PG | | 148 (10.2) |
|  | PhD | | 28 (1.9) |
| Socioeconomic status | Upper | | 356 (27.4) |
|  | Upper Middle | | 486 (37.4) |
|  | Lower Middle | | 264 (20.3) |
|  | Lower | | 192 (14.8) |
| Religion | Hinduism | | 1228 (84.5) |
|  | Islam | | 151 (10.4) |
|  | Others^α^ | | 74 (5.1) |
| Social categories | Unreserved | | 866 (67.0) |
|  | OBC | | 259 (20.0) |
|  | SC | | 137 (10.6) |
|  | ST | | 31 (2.4) |

N= count; UG= undergraduate; PG= post graduate; ^α^Others= Christianity, Sikhism, Jainism, Buddhism, Sanamahism, and those unaffiliated or not disclosed; OBC= other backward classes; SC= scheduled castes; ST= scheduled tribes

Supplementary Table 2: Regression analysis for anthropometric and blood pressure measures as dependent variables and PCE score as independent variable

| Dependent variable | R | R^2^ | β (unstandardized) | Std. Error | Beta (Standardized) | t | p-value |
| --- | --- | --- | --- | --- | --- | --- | --- |
| BMI^a^ | 0.355 | 0.126 | -0.233 | 0.074 | -0.086 | -3.151 | 0.002* |
| WC^a^ | 0.462 | 0.213 | -0.595 | 0.177 | -0.087 | -3.355 | 0.001* |
| WHR^a^ | 0.438 | 0.192 | -0.002 | 0.001 | -0.049 | -1.867 | 0.062 |
| WHtR^a^ | 0.342 | 0.117 | -0.004 | 0.001 | -0.089 | -3.227 | 0.001* |
| SBP^b^ | 0.461 | 0.212 | 0.001 | 0.184 | 0.000 | 0.004 | 0.997 |
| DBP^b^ | 0.100 | 0.010 | 0.115 | 0.147 | 0.022 | 0.785 | 0.433 |

*Significant at p-value<0.05; BMI= Body Mass Index; WC= Waist Circumference; WHR= Waist-to-Hip Ratio; WHtR= Waist-to-Height Ratio; SBP= Systolic Blood Pressure; DBP= Diastolic Blood Pressure; ^a^adjusted for age, sex, education, socioeconomic status, and social category; ^b^adjusted for age, sex, education, socioeconomic status; R= correlation coefficient; R^2^= coefficient of determination; β = regression coefficient; t= t-statistic

Supplementary Table 3: Distribution of general and central obesity across groups exposed and unexposed to different PCEs items

| PCE items |  | BMI | | | WC | | WHR | | WHtR | |
| --- | --- | --- | --- | --- | --- | --- | --- | --- | --- | --- |
|  |  | Normal | Underweight | Overweight/ obese | Normal | High | Normal | High | Normal | High |
|  |  | N (%) | N (%) | N (%) | N (%) | N (%) | N (%) | N (%) | N (%) | N (%) |
| Did you have at least one caregiver with whom you felt safe? | No | 50 (34) | 26 (17.7) | 71 (48.3) | 107 (72.8) | 40 (27.2) | 116 (78.9) | 31 (21.1) | 90 (61.2) | 57 (38.8) |
|  | Yes | 516 (39.7) | 252 (19.4) | 533 (41) | 973 (75.1) | 323 (24.9) | 1004 (77.5) | 291 (22.5) | 868 (67.1) | 426 (32.9) |
|  | p | 0.226 | | | 0.545 | | 0.703 | | 0.154 | |
| Did you have at least one good friend? | No | 60 (37) | 28 (17.3) | 74 (45.7) | 116 (71.6) | 46 (28.4) | 122 (75.3) | 40 (24.7) | 106 (65.8) | 55 (34.2) |
|  | Yes | 506 (39.3) | 250 (19.4) | 530 (41.2) | 964 (75.3) | 317 (24.7) | 998 (78) | 282 (22) | 852 (66.6) | 428 (33.4) |
|  | p | 0.540 | | | 0.313 | | 0.444 | | 0.854 | |
| Did you have ‘beliefs’ that gave you comfort? | No | 57 (35) | 28 (17.2) | 78 (47.9) | 110 (67.1) | 54 (32.9) | 127 (77.4) | 37 (22.6) | 103 (63.2) | 60 (36.8) |
|  | Yes | 509 (39.6) | 250 (19.5) | 526 (40.9) | 970 (75.8) | 309 (24.2) | 993 (77.7) | 285 (22.3) | 855 (66.9) | 423 (33.1) |
|  | p | 0.241 | | | 0.015^*^ | | 0.940 | | 0.345 | |
| Did you have enjoyment at school? | No | 55 (34.2) | 19 (11.8) | 87 (54) | 107 (66.5) | 54 (33.5) | 124 (77) | 37 (23) | 99 (61.5) | 62 (38.5) |
|  | Yes | 511 (39.7) | 259 (20.1) | 517 (40.2) | 973 (75.9) | 309 (24.1) | 996 (77.8) | 285 (22.2) | 859 (67.1) | 421 (32.9) |
|  | p | 0.002^*^ | | | 0.009^*^ | | 0.833 | | 0.155 | |
| Did you have at least one teacher that cared? | No | 73 (39.5) | 34 (18.4) | 78 (42.2) | 137 (74.1) | 48 (25.9) | 137 (74.1) | 48 (25.9) | 126 (68.1) | 59 (31.9) |
|  | Yes | 493 (39) | 244 (19.3) | 526 (41.6) | 943 (75) | 315 (25) | 983 (78.2) | 274 (21.8) | 832 (66.2) | 424 (33.8) |
|  | p | 0.955 | | | 0.791 | | 0.206 | | 0.616 | |
| Did you have good neighbours? | No | 122 (37.2) | 56 (17.1) | 150 (45.7) | 240 (73.8) | 85 (26.2) | 243 (74.8) | 82 (25.2) | 214 (65.8) | 111 (34.2) |
|  | Yes | 444 (39.6) | 222 (19.8) | 454 (40.5) | 840 (75.1) | 278 (24.9) | 877 (78.5) | 240 (21.5) | 744 (66.7) | 372 (33.3) |
|  | p | 0.220 | | | 0.638 | | 0.154 | | 0.783 | |
| Did you have an adult in your life (other than the person from question 1) who could provide you with support or advice? | No | 96 (38.7) | 52 (21) | 100 (40.3) | 184 (73.9) | 65 (26.1) | 192 (77.1) | 57 (22.9) | 168 (67.7) | 80 (32.3) |
|  | Yes | 470 (39.2) | 226 (18.8) | 504 (42) | 896 (75) | 298 (25) | 928 (77.8) | 265 (22.2) | 790 (66.2) | 403 (33.8) |
|  | p | 0.727 | | | 0.705 | | 0.815 | | 0.644 | |
| Did you have opportunities to have a good time? | No | 41 (37.6) | 20 (18.3) | 48 (44) | 84 (77.1) | 25 (22.9) | 81 (74.3) | 28 (25.7) | 76 (69.7) | 33 (30.3) |
|  | Yes | 525 (39.2) | 258 (19.3) | 556 (41.5) | 996 (74.7) | 338 (25.3) | 1039 (77.9) | 294 (22.1) | 882 (66.2) | 450 (33.8) |
|  | p | 0.877 | | | 0.578 | | 0.381 | | 0.456 | |
| Did you like yourself or felt comfortable with yourself? | No | 50 (29.9) | 20 (12) | 97 (58.1) | 108 (64.7) | 59 (35.3) | 115 (68.9) | 52 (31.1) | 95 (56.9) | 72 (43.1) |
|  | Yes | 516 (40.3) | 258 (20.1) | 507 (39.6) | 972 (76.2) | 304 (23.8) | 1005 (78.8) | 270 (21.2) | 863 (67.7) | 411 (32.3) |
|  | p | <0.001^*^ | | | 0.001^*^ | | 0.004^*^ | | 0.005^*^ | |
| Did you have predictable home routine, like regular meals and a regular bedtime? | No | 116 (36.9) | 64 (20.4) | 134 (42.7) | 236 (74.9) | 79 (25.1) | 245 (77.8) | 70 (22.2) | 213 (67.8) | 101 (32.2) |
|  | Yes | 450 (39.7) | 214 (18.9) | 470 (41.4) | 844 (74.8) | 284 (25.2) | 875 (77.6) | 252 (22.4) | 745 (66.1) | 382 (33.9) |
|  | p | 0.653 | | | 0.972 | | 0.959 | | 0.566 | |

*Significant at p-value<0.05; p= p-value; N= count; %=row wise percentage; BMI= Body Mass Index; WC= Waist Circumference; WHR= Waist-to-Hip Ratio; WHtR= Waist-to-Height Ratio

Supplementary Table 4: Distribution of hypertension across groups exposed and unexposed to different PCEs items

| PCE items |  | BP | | | |
| --- | --- | --- | --- | --- | --- |
|  |  | Normal | Elevated | HTN Stage 1 | HTN Stage 2 |
|  |  | N (%) | N (%) | N (%) | N (%) |
| Did you have at least one caregiver with whom you felt safe? | No | 88 (61.1) | 10 (6.9) | 38 (26.4) | 8 (5.6) |
|  | Yes | 789 (61.4) | 95 (7.4) | 307 (23.9) | 95 (7.4) |
|  | p | 0.806 | | | |
| Did you have at least one good friend? | No | 102 (63.4) | 9 (5.6) | 41 (25.5) | 9 (5.6) |
|  | Yes | 775 (61.1) | 96 (7.6) | 304 (24) | 94 (7.4) |
|  | p | 0.644 | | | |
| Did you have ‘beliefs’ that gave you comfort? | No | 98 (60.9) | 13 (8.1) | 39 (24.2) | 11 (6.8) |
|  | Yes | 779 (61.4) | 92 (7.2) | 306 (24.1) | 92 (7.2) |
|  | p | 0.982 | | | |
| Did you have enjoyment at school? | No | 107 (66.9) | 10 (6.3) | 35 (21.9) | 8 (5) |
|  | Yes | 770 (60.6) | 95 (7.5) | 310 (24.4) | 95 (7.5) |
|  | p | 0.425 | | | |
| Did you have at least one teacher that cared? | No | 118 (64.5) | 10 (5.5) | 43 (23.5) | 12 (6.6) |
|  | Yes | 759 (60.9) | 95 (7.6) | 302 (24.2) | 91 (7.3) |
|  | p | 0.681 | | | |
| Did you have good neighbours? | No | 209 (64.9) | 20 (6.2) | 73 (22.7) | 20 (6.2) |
|  | Yes | 668 (60.3) | 85 (7.7) | 272 (24.5) | 83 (7.5) |
|  | p | 0.471 | | | |
| Did you have an adult in your life (other than the person from question 1) who could provide you with support or advice? | No | 167 (67.9) | 14 (5.7) | 54 (22) | 11 (4.5) |
|  | Yes | 710 (60) | 91 (7.7) | 291 (24.6) | 92 (7.8) |
|  | p | 0.077 | | | |
| Did you have opportunities to have a good time? | No | 75 (68.2) | 8 (7.3) | 23 (20.9) | 4 (3.6) |
|  | Yes | 802 (60.8) | 97 (7.3) | 322 (24.4) | 99 (7.5) |
|  | p | 0.317 | | | |
| Did you like yourself or felt comfortable with yourself? | No | 114 (68.3) | 11 (6.6) | 30 (18) | 12 (7.2) |
|  | Yes | 763 (60.4) | 94 (7.4) | 315 (24.9) | 91 (7.2) |
|  | p | 0.203 | | | |
| Did you have predictable home routine, like regular meals and a regular bedtime? | No | 191 (61.6) | 21 (6.8) | 83 (26.8) | 15 (4.8) |
|  | Yes | 686 (61.3) | 84 (7.5) | 262 (23.4) | 88 (7.9) |
|  | p | 0.221 | | | |

*Significant at p-value<0.05; p= p-value; N= count; %=row wise percentage; BP= blood pressure; HTN= hypertension

Supplementary Table 5: Prevalence of obesity and hypertension in ACE subcategories stratified for PCE levels and PCE subcategories stratified for ACE levels

| Physical health outcomes | | | 0-5 PCEs | | | 6-9 PCEs | | | 10 PCEs | | |
| --- | --- | --- | --- | --- | --- | --- | --- | --- | --- | --- | --- |
|  |  |  | 0 ACE | 1-3 ACEs | ≥4 ACEs | 0 ACE | 1-3 ACEs | ≥4 ACEs | 0 ACE | 1-3 ACEs | ≥4 ACEs |
|  |  |  | N (%) | N (%) | N (%) | N (%) | N (%) | N (%) | N (%) | N (%) | N (%) |
| BMI | Normal | | 1 (14.3) | 22 (41.5) | 13 (28.3) | 66 (39.8) | 168 (40.4) | 42 (30) | 84 (38.7) | 138 (41.3) | 32 (46.4) |
|  | Underweight | | 2 (28.6) | 6 (11.3) | 1 (2.2) | 43 (25.9) | 83 (20) | 31 (22.1) | 49 (22.6) | 53 (15.9) | 10 (14.5) |
|  | Overweight/obese | | 4 (57.1) | 25 (47.2) | 32 (69.6) | 57 (34.3) | 165 (39.7) | 67 (47.9) | 84 (38.7) | 143 (42.8) | 27 (39.1) |
|  | p-value | | 0.036* | | | 0.074 | | | 0.267 | | |
| WC | Normal | | 3 (42.9) | 35 (66) | 28 (60.9) | 139 (83.7) | 320 (76.9) | 97 (69.8) | 158 (73.1) | 246 (73.9) | 54 (80.6) |
|  | High | | 4 (57.1) | 18 (34) | 18 (39.1) | 27 (16.3) | 96 (23.1) | 42 (30.2) | 58 (26.9) | 87 (26.1) | 13 (19.4) |
|  | p-value | | 0.477 | | | 0.015* | | | 0.455 | | |
| WHR | Normal | | 5 (71.4) | 37 (69.8) | 34 (73.9) | 131 (78.9) | 315 (75.7) | 108 (77.7) | 182 (84.3) | 254 (76.3) | 54 (81.8) |
|  | High | | 2 (28.6) | 16 (30.2) | 12 (26.1) | 35 (21.1) | 101 (24.3) | 31 (22.3) | 34 (15.7) | 79 (23.7) | 12 (18.2) |
|  | p-value | | 0.903 | | | 0.687 | | | 0.068 | | |
| WHtR | Normal | | 4 (57.1) | 33 (62.3) | 27 (58.7) | 119 (72.1) | 282 (68) | 85 (61.2) | 143 (66.2) | 217 (65.2) | 48 (71.6) |
|  | High | | 3 (42.9) | 20 (37.7) | 19 (41.3) | 46 (27.9) | 133 (32) | 54 (38.8) | 73 (33.8) | 116 (34.8) | 19 (28.4) |
|  | p-value | | 0.921 | | | 0.122 | | | 0.593 | | |
| BP | Normal | | 3 (42.9) | 40 (76.9) | 31 (67.4) | 88 (54.3) | 250 (60.5) | 96 (69.6) | 123 (57.5) | 197 (59.5) | 49 (73.1) |
|  | Elevated | | 0 (0) | 2 (3.8) | 4 (8.7) | 11 (6.8) | 28 (6.8) | 8 (5.8) | 11 (5.1) | 32 (9.7) | 9 (13.4) |
|  | HTN Stage 1 | | 4 (57.1) | 8 (15.4) | 8 (17.4) | 55 (34) | 109 (26.4) | 26 (18.8) | 56 (26.2) | 75 (22.7) | 4 (6) |
|  | HTN Stage 2 | | 0 (0) | 2 (3.8) | 3 (6.5) | 8 (4.9) | 26 (6.3) | 8 (5.8) | 24 (11.2) | 27 (8.2) | 5 (7.5) |
|  | p-value | | 0.171 | | | 0.133 | | | 0.005* | | |
|  | | | 0 ACE | | | 1-3 ACEs | | | ≥4 ACEs | | |
|  |  |  | 0-5 PCEs | 6-9 PCEs | 10 PCEs | 0-5 PCEs | 6-9 PCEs | 10 PCEs | 0-5 PCEs | 6-9 PCEs | 10 PCEs |
| BMI | | Normal | 1 (14.3) | 66 (39.8) | 84 (38.7) | 22 (41.5) | 168 (40.4) | 138 (41.3) | 13 (28.3) | 42 (30) | 32 (46.4) |
|  |  | Underweight | 2 (28.6) | 43 (25.9) | 49 (22.6) | 6 (11.3) | 83 (20) | 53 (15.9) | 1 (2.2) | 31 (22.1) | 10 (14.5) |
|  |  | Overweight/ obese | 4 (57.1) | 57 (34.3) | 84 (38.7) | 25 (47.2) | 165 (39.7) | 143 (42.8) | 32 (69.6) | 67 (47.9) | 27 (39.1) |
|  |  | p-value | 0.581 | | | 0.409 | | | 0.001* | | |
| WC | | Normal | 3 (42.9) | 139 (83.7) | 158 (73.1) | 35 (66) | 320 (76.9) | 246 (73.9) | 28 (60.9) | 97 (69.8) | 54 (80.6) |
|  |  | High | 4 (57.1) | 27 (16.3) | 58 (26.9) | 18 (34) | 96 (23.1) | 87 (26.1) | 18 (39.1) | 42 (30.2) | 13 (19.4) |
|  |  | p-value | 0.005* | | | 0.191 | | | 0.067 | | |
| WHR | | Normal | 5 (71.4) | 131 (78.9) | 182 (84.3) | 37 (69.8) | 315 (75.7) | 254 (76.3) | 34 (73.9) | 108 (77.7) | 54 (81.8) |
|  |  | High | 2 (28.6) | 35 (21.1) | 34 (15.7) | 16 (30.2) | 101 (24.3) | 79 (23.7) | 12 (26.1) | 31 (22.3) | 12 (18.2) |
|  |  | p-value | 0.316 | | | 0.592 | | | 0.601 | | |
| WHtR | | Normal | 4 (57.1) | 119 (72.1) | 143 (66.2) | 33 (62.3) | 282 (68) | 217 (65.2) | 27 (58.7) | 85 (61.2) | 48 (71.6) |
|  |  | High | 3 (42.9) | 46 (27.9) | 73 (33.8) | 20 (37.7) | 133 (32) | 116 (34.8) | 19 (41.3) | 54 (38.8) | 19 (28.4) |
|  |  | p-value | 0.377 | | | 0.582 | | | 0.259 | | |
| BP | | Normal | 3 (42.9) | 88 (54.3) | 123 (57.5) | 40 (76.9) | 250 (60.5) | 197 (59.5) | 31 (67.4) | 96 (69.6) | 49 (73.1) |
|  |  | Elevated | 0 (0) | 11 (6.8) | 11 (5.1) | 2 (3.8) | 28 (6.8) | 32 (9.7) | 4 (8.7) | 8 (5.8) | 9 (13.4) |
|  |  | HTN Stage 1 | 4 (57.1) | 55 (34) | 56 (26.2) | 8 (15.4) | 109 (26.4) | 75 (22.7) | 8 (17.4) | 26 (18.8) | 4 (6) |
|  |  | HTN Stage 2 | 0 (0) | 8 (4.9) | 24 (11.2) | 2 (3.8) | 26 (6.3) | 27 (8.2) | 3 (6.5) | 8 (5.8) | 5 (7.5) |
|  |  | p-value | 0.134 | | | 0.131 | | | 0.196 | | |

*Significant at p-value<0.05; N=Count; %= column percentage; ACE= adverse childhood experience; PCE= positive childhood experience; Mod/Sev =moderate/severe; BMI= Body Mass Index; WC= Waist Circumference; WHR= Waist-to-Hip Ratio; WHtR= Waist-to-Height Ratio; BP= Blood Pressure; HTN= hypertension

Supplementary Table 6: Overall and stratified correlation of ACE and PCE score with depression, anxiety, stress, and well-being scores

| Stratification | Independent variable | BMI | WC | WHR | WHtR | SBP | DBP |
| --- | --- | --- | --- | --- | --- | --- | --- |
| None | ACE Score | 0.096^**^ | 0.090^**^ | 0.048^*^ | 0.084^**^ | -0.039 | -0.049^*^ |
| None | PCE Score | -0.077^**^ | -0.068^**^ | -0.034 | -0.082^**^ | 0.037 | 0.043 |
| 0-5 PCEs | ACE Score | 0.102 | 0.060 | 0.045 | 0.087 | -0.003 | 0.065 |
| 6-9 PCEs | ACE Score | 0.153^**^ | 0.128^**^ | 0.045 | 0.109^**^ | -0.019 | -0.033 |
| 10 PCEs | ACE Score | 0.024 | 0.012 | 0.015 | 0.002 | -0.038 | -0.118^**^ |
| 0 ACEs | PCE Score | 0.012 | -0.007 | -0.013 | -0.013 | 0.041 | 0.057 |
| 1-3 ACEs | PCE Score | -0.004 | 0.003 | 0.004 | -0.012 | 0.041 | 0.027 |
| ≥4 ACEs | PCE Score | -0.248^**^ | -0.224^**^ | -0.129^*^ | -0.257^**^ | -0.023 | -0.030 |

**Significant at p-value<0.01; *Significant at p-value<0.05; ACE= adverse childhood experience; PCE= positive childhood experience; BMI= Body Mass Index; WC= Waist Circumference; WHR= Waist-to-Hip Ratio; WHtR= Waist-to-Height Ratio; SBP= Systolic Blood Pressure; DBP= Diastolic Blood Pressure
